# Dietary *Sporolactobacillus laevolacticus* Improves Growth Performance, Intestinal Health, and Immune-Antioxidant Related Responses in Juvenile Coho Salmon (*Oncorhynchus kisutch*)

**DOI:** 10.3390/microorganisms14061367

**Published:** 2026-06-20

**Authors:** Qin Zhang, Lan Li, Xin Guo, Yuping Xia, Shanping Xiong, Xinjing Wei, Rongkai Zhu, Weiguang Kong, Yongqiang Liu, Tong Tong

**Affiliations:** 1 Guangxi Key Laboratory for Polysaccharide Materials and Modifications, Guangxi Marine Microbial Resources Industrialization Engineering Technology Research Center, School of Marine Sciences and Biotechnology, Guangxi Minzu University, 158 University West Road, Nanning 530008, China; zhangqin@gxmzu.edu.cn (Q.Z.); lilan@stu.gxmzu.edu.cn (L.L.); guoxin@stu.gxmzu.edu.cn (X.G.); xiayuping@stu.gxmzu.edu.cn (Y.X.); xiongshanping@stu.gxmzu.edu.cn (S.X.); weixinjing@stu.gxmzu.edu.cn (X.W.); zhurongkai@stu.gxmzu.edu.cn (R.Z.); 2 Key Laboratory of Breeding Biotechnology and Sustainable Aquaculture, Institute of Hydrobiology, Chinese Academy of Sciences, 7 Donghu South Road, Wuhan 430072, China; kongweiguang@ihb.ac.cn

**Keywords:** probiotic, gut microbiota, intestinal morphology, antioxidant capacity, inflammatory response

## Abstract

Probiotics are considered promising feed additives for enhancing fish health and production performance in aquaculture. This study evaluated the effects of dietary supplementation with *Sporolactobacillus laevolacticus* on growth performance, feed utilization, intestinal health, and physiological responses in juvenile coho salmon (*Oncorhynchus kisutch*). Fish were fed a control diet or diets supplemented with *S. laevolacticus* at 0.89 × 10^7^, 0.90 × 10^9^, or 0.87 × 10^11^ CFU/g for 10 weeks. Compared with the control, *S. laevolacticus* supplementation significantly increased final body weight, weight gain rate, specific growth rate, and protein efficiency ratio, while decreasing the feed conversion ratio (*p* < 0.05). It also significantly enhanced intestinal protease and α-amylase activities, improved serum biochemical and immune-related parameters, and promoted better intestinal morphology (*p* < 0.05). Additionally, *S. laevolacticus* supplementation led to elevated expression of antioxidant-related genes, reduced expression of pro-inflammatory genes, and altered gut microbial composition, characterized by a decrease in Proteobacteria and increases in Firmicutes and *Lactobacillales*. Among the tested dosages, 0.90 × 10^9^ CFU/g produced the most consistent improvements in growth performance, digestive function, intestinal health, antioxidant and immune responses, and gut microbial composition, and was therefore identified as the optimal supplementation level. Collectively, dietary *S. laevolacticus* at 0.90 × 10^9^ CFU/g improved growth performance and intestinal health in juvenile coho salmon, highlighting its potential as a probiotic candidate for coho salmon aquaculture.

## 1. Introduction

Coho salmon (*Oncorhynchus kisutch*) is rich in high-quality protein and n-3 polyunsaturated fatty acids, giving it substantial economic and nutritional value among cold-water aquaculture species. As a result, it has become one of the major farmed species in the global high-end seafood market [1,2]. In recent years, however, the expansion of coho salmon farming toward larger-scale and more intensive production has been accompanied by increasingly prominent challenges, including growth suppression, elevated oxidative stress, disruption of immune homeostasis, and impaired intestinal function caused by high stocking density, environmental fluctuations, and pathogen exposure. These problems have become important constraints on healthy production and the sustainable development of the industry [3]. In recent years, the global production of farmed coho salmon has continued to increase, accompanied by growing market demand and economic value, making it one of the most important cold-water aquaculture species worldwide [4]. Meanwhile, bacterial diseases caused by pathogens such as Aeromonas spp. and other opportunistic bacteria, together with environmental stress associated with intensive farming practices, have become important constraints affecting the health and productivity of cultured coho salmon [5]. Although antibiotics and chemical therapeutics can reduce disease risk to some extent, their long-term use is associated with antimicrobial resistance, drug residues, and environmental contamination. Accordingly, the development of safe, environmentally friendly, and effective alternatives has become a major focus in modern aquaculture nutrition and disease control research [6,7].

Probiotics have been widely investigated in aquaculture because they can modulate the intestinal microecology, improve nutrient utilization, enhance immune defense, and alleviate farming-related stress, and are therefore regarded as promising alternatives to antibiotics [8,9,10]. Previous studies have shown that dietary supplementation with probiotics such as *Bacillus cereus* and *Bacillus pumilus* can improve growth performance in coho salmon while enhancing antioxidant capacity, immune-related indices, and intestinal structural integrity [11]. Similarly, functional immunostimulants such as yeast β-glucan have also been reported to promote growth, improve intestinal barrier function, and enhance host health by modulating the gut microbiota and related metabolic processes [5]. Nevertheless, probiotic effects are highly strain-dependent, and marked differences often exist among strains in host adaptability, metabolic characteristics, and regulatory patterns. Therefore, identifying probiotic candidates that combine processing stability, intestinal colonization potential, and biological activity remains an important task in aquaculture probiotic research.

*Sporolactobacillus laevolacticus* is a distinctive probiotic taxon characterized by both lactic acid-producing activity and spore-forming capacity [12]. Compared with conventional non-spore-forming lactic acid bacteria, members of this group generally exhibit greater survival and stability under high-temperature processing, gastric acidity, bile salts, and other complex environmental stresses [13]. Previous studies have shown that *Sporolactobacillus* species can produce lactic acid, exopolysaccharides, and other bioactive metabolites, suggesting potential value in maintaining gut microbial homeostasis, improving the intestinal microenvironment, and promoting host health [14,15]. From an applied perspective, these bacteria combine the environmental resilience typical of spore-forming microorganisms with the metabolically active features of lactic acid bacteria, indicating promising prospects for functional feed development and use under challenging aquaculture conditions [16,17]. In addition, members of the genus Sporolactobacillus are capable of producing organic acids and other bioactive metabolites that may contribute to the suppression of undesirable microorganisms and the maintenance of intestinal microbial balance. Compared with conventional Bacillus probiotics, *S. laevolacticus* combines the environmental robustness associated with spore formation and the metabolic characteristics typical of lactic acid bacteria [13]. Therefore, this species may represent a complementary probiotic candidate that integrates the stability of Bacillus spp. with the functional attributes commonly associated with Lactobacillus spp. However, compared with more commonly studied aquaculture probiotics such as *Bacillus*, *Lactobacillus*, and *Clostridium butyricum*, research on *S. laevolacticus* in aquatic animals remains limited, and its nutritional and health-promoting effects in coho salmon have not yet been systematically characterized. To the best of our knowledge, information regarding the application of *S. laevolacticus* in aquaculture species remains extremely limited, and its potential benefits in coho salmon have not been investigated. Therefore, the present study was conducted to evaluate the effects of dietary *S. laevolacticus* supplementation on growth performance, intestinal health, immune and antioxidant responses, and gut microbial communities in juvenile coho salmon. The biological effects of probiotics on the host are typically multifaceted, involving growth performance, nutrient utilization, metabolic regulation, maintenance of intestinal structure, molecular responses, and reshaping of the gut microecological environment. Therefore, evaluation based on a single endpoint is often insufficient to fully characterize their biological functions [18,19,20]. In addition to host molecular responses, alterations in gut microbial community structure provide another important perspective for understanding the mechanisms underlying probiotic action. Previous studies have shown that probiotics may influence gut microbial composition and metabolic potential by competing for adhesion sites and nutrients, producing organic acids and other antimicrobial metabolites, and modulating the local intestinal microenvironment [21]. Accordingly, an integrated assessment combining growth performance, digestive function, antioxidant and immune-related indicators, together with gut microbiota profiling, is more informative for elucidating the overall effects of probiotics in aquatic animals and the pathways through which they act.

Based on this rationale, the present study used juvenile coho salmon as the experimental model to systematically evaluate the effects of dietary supplementation with different levels of *S. laevolacticus* on growth performance, nutrient utilization, intestinal digestive function, antioxidant status, immune-related responses, and gut microbial community structure. The findings of this study may provide a useful reference for the application of *S. laevolacticus* in healthy coho salmon culture and for the development of functional aquafeeds.

## 2. Materials and Methods

### 2.1. Strain Source, Cultivation, Identification, and Preliminary Biosafety Evaluation

The *S. laevolacticus* strain used in this study (strain No. GXF3206) was isolated from rhizosphere soil collected from the Maowei Sea mangrove area in Qinzhou, China. The strain was first activated on LB agar plates and incubated at 28 °C for 48 h. Subsequently, a single colony with uniform morphology was aseptically picked and inoculated into 10 mL of LB liquid medium, followed by shaking incubation at 28 °C and 140 r/min for 10 h to prepare the primary seed culture. The primary seed culture was then transferred into 500 mL of LB liquid medium at an inoculation rate of 1% (*v*/*v*) and incubated under the same conditions for an additional 48 h. After cultivation, viable bacterial counts were determined by the plate-counting method. The culture was stored at 4 °C for short-term use and used in subsequent experiments within 24 h.

To determine the taxonomic identity of the strain used in this study, molecular identification was performed. Genomic DNA was extracted from the strain, and the 16S rRNA gene was amplified by PCR using the universal primers 27F and 1492R. The purified PCR products were then sent to Sangon Biotech (Shanghai, China) for sequencing. The obtained sequences were compared against the EZBioCloud and NCBI BLAST databases to preliminarily determine the taxonomic affiliation of the strain. To obtain a more reliable sequence, the 16S rRNA amplicon was further subjected to T-A cloning, followed by colony PCR amplification using M13F/M13R primers. The amplified products were purified, resequenced, and assembled from the forward and reverse reads. Based on sequence similarity analysis between the assembled high-quality sequence and reference sequences retrieved from the databases, the strain was finally identified as *S. laevolacticus* and deposited in the Culture Collection of the Marine Biology Laboratory, Guangxi Minzu University (strain No. GXF3206).

To preliminarily evaluate the biosafety of strain GXF3206, hemolytic activity was assessed using the blood agar plate method according to established probiotic safety evaluation procedures [22]. The activated strain was streaked onto blood agar plates containing 5% defibrinated sheep blood and incubated at 37 °C for 24–48 h. The plates were then examined for the presence of clear or green hemolytic zones surrounding the colonies. No obvious transparent or green hemolytic ring was observed around the colonies of strain GXF3206, and the strain was therefore classified as γ-hemolytic, indicating the absence of detectable hemolytic activity and suggesting good preliminary biosafety.

Tolerance to simulated gastrointestinal conditions was evaluated according to the methods of Zhang et al. [23] and Liu et al. [24]. Briefly, 0.2 mL of the washed bacterial suspension was placed into a 2.0 mL screw-cap tube, followed by the addition of 1.0 mL of simulated gastric fluid (pH 2.0) or simulated intestinal fluid (pH 8.0) and 0.3 mL of 0.5% (*w*/*v*) NaCl solution to obtain a final volume of 1.5 mL. The mixture was vortexed thoroughly and incubated at 15 °C. Samples (0.1 mL) were collected after 3 h in simulated gastric fluid and after 4 h in simulated intestinal fluid for viable count determination. The 3 h survival rate in simulated gastric fluid (3-SR-SGF) and the 4 h survival rate in simulated intestinal fluid (4-SR-SIF) were then calculated. The results showed that the 3-SR-SGF and 4-SR-SIF values of strain GXF3206 were 36.81 ± 3.39% and 46.65 ± 5.71%, respectively, indicating its potential tolerance to gastrointestinal conditions.

To further assess the in vivo biosafety of this strain in juvenile coho salmon [25], bacterial suspensions of different concentrations (1 × 10^7^, 1 × 10^9^, and 1 × 10^11^ CFU/mL) were prepared using sterile physiological saline. A sterile saline control group and three bacterial suspension treatment groups were established. Each fish was intraperitoneally injected with 0.2 mL of the corresponding suspension. Healthy juvenile coho salmon with an average body weight of 128.78 ± 6.27 g were used, with 30 fish allocated to each treatment group, and the trial lasted for 7 d. During the experimental period, fish survival, feeding behavior, and external abnormalities were monitored daily. The results showed that the survival rate of juvenile coho salmon in all treatment groups was 100%, and no obvious abnormal behavior or external lesions were observed, suggesting that the strain did not produce apparent acute adverse effects on juvenile coho salmon within the tested dosage range and could therefore be used in the subsequent feeding trial.

### 2.2. Preparation of Experimental Diets

The basal diet used in this study was purchased from Shandong Kangkairun Marine Technology Co., Ltd. (Weifang, China). All ingredients met feed-grade standards, and the dietary formulation and proximate composition are presented in Table 1. The probiotic inclusion levels were determined with reference to previous feeding studies in juvenile coho salmon and in consideration of the safety evaluation results obtained in the present study. Previous reports have shown that dietary supplementation levels of *Lactobacillus plantarum* in coho salmon ranged from 10^5^ to 10^9^ CFU/g diet, whereas *Bacillus pumilus* was applied at 2 × 10^6^ to 8 × 10^10^ CFU/g diet. In addition, *Bacillus cereus* exhibited favorable growth-promoting and health-improving effects at 4 × 10^9^ CFU/g diet. Based on these reported ranges and the in vivo safety assessment of the present strain, four experimental groups were established by supplementing the basal diet with different levels of *S. laevolacticus*: a control group (CK, without probiotic supplementation), a low-dose group (GL, bacterial suspension at 1 × 10^7^ CFU/g), a medium-dose group (GM, bacterial suspension at 1 × 10^9^ CFU/g), and a high-dose group (GH, bacterial suspension at 1 × 10^11^ CFU/g).

During diet preparation, bacterial suspensions of *S. laevolacticus* at the corresponding concentrations were directly added to the basal diet ingredients at predetermined proportions, thoroughly mixed, and used to prepare the experimental diets. An equal volume of sterile physiological saline was added to the control diet to eliminate the potential effect of liquid addition itself. All experimental diets were prepared freshly on the day of use and temporarily stored at 4 °C after preparation. The amount of bacterial suspension added was determined through preliminary trials, and the actual viable bacterial counts in each diet were measured by the plate-counting method. The viable bacterial counts of the experimental diets are shown in Table 2, and all subsequent dosage calculations were based on the actual bacterial counts in the diets. The viable counts were determined immediately after diet preparation. Because the diets were freshly prepared and used within a short period under refrigerated conditions, bacterial viability during long-term storage was not monitored in the present study.

### 2.3. Rearing Management and Experimental Design

Juvenile coho salmon used in this study were obtained from the Rainbow Trout Breeding Farm located in Nanfen District, Benxi City, China, with an initial body weight of 128.78 ± 6.27 g. Before the start of the feeding trial, the culture tanks and the inner surfaces of the net cages were thoroughly disinfected with 10 mg/L potassium permanganate solution. All fish were acclimated for 14 days before the formal experiment to adapt them to the rearing conditions and the basal diet.

During the acclimation period, fish were reared under a natural flow-through water system. The water temperature ranged from 10 to 18 °C, dissolved oxygen remained at ≥6.0 mg/L, the water exchange rate was maintained at ≥100 L/s, the surface water velocity was ≥2 cm/s, and pH was maintained between 7.5 and 7.8. Fish were exposed to a natural photoperiod throughout the acclimation period. During this period, the fish were fed the basal diet three times daily (08:00, 12:00, and 16:00). The feeding ration was adjusted according to actual feed intake, and feeding was stopped when the fish ceased active feeding. The daily feed intake of each net cage was recorded.

After the acclimation period, all fish were fasted for 24 h. A total of 240 healthy juvenile coho salmon were then randomly assigned to four treatment groups, each with three replicates, giving a total of 12 net cages (1.0 × 1.0 × 0.8 m, length × width × height), with 20 fish stocked per cage. The positions of the cages within the culture system were randomly assigned before the start of the experiment. All cages were maintained under the same flow-through water conditions, and water temperature, dissolved oxygen, and pH were monitored routinely throughout the feeding trial to ensure environmental consistency among treatments. Fish in the respective groups were fed the experimental diets corresponding to the CK, GL, GM, and GH treatments. The formal feeding trial was conducted under the same environmental conditions as those used during acclimation and lasted for 10 weeks.

### 2.4. Sample Collection and Processing

At the end of the 10-week feeding trial, all fish were fasted for 24 h prior to sampling. Juvenile coho salmon were anesthetized with 40 mg/L tricaine methanesulfonate (MS-222), after which body weight and body length were measured individually for the calculation of growth performance indices.

Six fish were randomly selected from each net cage for sample collection. Blood was collected aseptically from the caudal vein using disposable sterile syringes. The blood samples were transferred into sterile 2 mL centrifuge tubes and allowed to clot, followed by centrifugation at 3000× *g* and 4 °C for 15 min. The supernatant serum was then carefully collected, transferred into new sterile tubes, and stored at −80 °C for subsequent serum biochemical analyses. All statistical analyses were performed using the net cage as the experimental unit. For parameters measured from multiple fish within the same cage, individual observations were averaged to generate a single cage mean prior to statistical analysis.

After blood collection, the fish were dissected under aseptic conditions, and the intestinal tissues were rapidly isolated. Intestinal tissue samples from three fish were immediately frozen in liquid nitrogen and then stored at −80 °C for gene expression analysis. In addition, intestinal contents from another three fish were rapidly frozen in liquid nitrogen and stored at −80 °C for gut microbiota analysis. At the same time, foregut, midgut, and hindgut tissues from each sampled fish were fixed in 10% neutral formalin for histological sectioning and morphological analysis.

### 2.5. Calculation of Growth Performance

The weight gain rate (WGR), specific growth rate (SGR), daily growth index (DGI), feed conversion ratio (FCR), protein efficiency ratio (PER), condition factor (CF), hepatosomatic index (HSI), viscera index (VSI), and survival rate (SR) of juvenile coho salmon were calculated using the following formula:

$$\mathrm{WGR}\;(\%)\;=\;100\;\times \;\frac{\mathrm{final}\;\mathrm{body}\;\mathrm{weight}\;(g)-\mathrm{initial}\;\mathrm{body}\;\mathrm{weight}\;(g)}{\mathrm{initial}\;\mathrm{body}\;\mathrm{weight}\;(g)}$$

$$\mathrm{SGR}\;(\%/d)=100\;\times \;\frac{[\ln\left(\mathrm{final}\;\mathrm{body}\;\mathrm{weight})\;(g)\right]-[\ln\left(\mathrm{initial}\;\mathrm{body}\;\mathrm{weight})\;(g)\right]}{\mathrm{days}}$$

$$\mathrm{DGI}\;(\%/d)=100\;\times \;\frac{[{\left(\mathrm{final}\;\mathrm{body}\;\mathrm{weight})\;(g)\right]}^{1/3}-[{\left(\mathrm{initial}\;\mathrm{body}\;\mathrm{weight})\;(g)\right]}^{1/3}\;}{\mathrm{days}}$$

$$\mathrm{FCR}=\frac{\mathrm{total}\;\mathrm{feed}\;\mathrm{intake}\;(g)}{\mathrm{final}\;\mathrm{body}\;\mathrm{weight}\;(g)-\mathrm{initial}\;\mathrm{body}\;\mathrm{weight}\;(g)}$$

$$\mathrm{PER}=\frac{\mathrm{final}\;\mathrm{body}\;\mathrm{weight}\;(g)-\mathrm{initial}\;\mathrm{body}\;\mathrm{weight}\;(g)}{\mathrm{total}\;\mathrm{feed}\;\mathrm{protein}\;\mathrm{intake}\;(g)}$$

$$\mathrm{CF}\;(\%)=100\;\times \;\frac{\mathrm{body}\;\mathrm{weight}\;(g)}{{\left[\mathrm{body}\;\mathrm{length}\;(\mathrm{cm})\right]}^{3}}$$

$$\mathrm{HSI}\;(\%)=100\;\times \;\frac{\mathrm{liver}\;\mathrm{weight}\;(g)\;}{\mathrm{body}\;\mathrm{weight}\;(g)\;}$$

$$\mathrm{VSI}\;(\%)=100\;\times \;\frac{\mathrm{viscera}\;\mathrm{weight}\;(g)\;}{\mathrm{body}\;\mathrm{weight}\;(g)}$$

$$\mathrm{SR}\;(\%)=100\;\times \;\frac{\mathrm{final}\;\mathrm{number}\;\mathrm{of}\;\mathrm{fish}}{\mathrm{initial}\;\mathrm{number}\;\mathrm{of}\;\mathrm{fish}}$$

For growth performance calculations, biomass gain and feed utilization parameters were corrected for mortality when applicable.

### 2.6. Determination of Serum Biochemical Indices

Serum biochemical parameters were determined using commercial assay kits purchased from Nanjing Jiancheng Bioengineering Institute (Nanjing, China), and all procedures were performed in strict accordance with the manufacturers’ instructions.

The measured indices included glucose (GLU; catalog no. A154-1-1), high-density lipoprotein cholesterol (HDL-C; catalog no. A112-1-1), low-density lipoprotein cholesterol (LDL-C; catalog no. A113-1-1), albumin (ALB; catalog no. A028-2-1), lysozyme (LZM; catalog no. A050-1-1), acid phosphatase (ACP; catalog no. A060-2-1), alkaline phosphatase (AKP; catalog no. A059-2), total protein (TP; catalog no. A045-2), triglycerides (TG; catalog no. A110-1-1), and total cholesterol (T-CHO; catalog no. A111-1-1).

GLU was measured by the glucose oxidase method and expressed as mmol/L. HDL-C and LDL-C were determined by the direct method and expressed as mmol/L. ALB was measured by the bromocresol green method and expressed as g/L. TP was determined by the Coomassie brilliant blue method and expressed as mg/mL. TG and T-CHO were measured by enzymatic methods and expressed as mmol/L. LZM was determined by the turbidimetric method and expressed as μg/mL. ACP and AKP were both measured by the microplate method and expressed as U/mL.

All indices were measured and calculated according to the instructions provided with the respective assay kits.

### 2.7. Determination of Intestinal Digestive Enzyme Activities

Intestinal digestive enzyme activities were determined according to the method of Zhang et al. [26], using commercial assay kits obtained from Nanjing Jiancheng Bioengineering Institute (Nanjing, China). The measured indices included protease (catalog no. A080-1-1), α-amylase (AMS, catalog no. C016-1-1), and lipase (LPS, catalog no. A054-2-1). All assays were performed strictly in accordance with the manufacturer’s instructions. Enzyme activities were normalized to the total protein content of each sample, and the results were expressed as enzyme activity units per milligram of protein (U/mg prot). Specifically, α-amylase activity was determined by a colorimetric method, and one unit of enzyme activity was defined as the amount of enzyme required to hydrolyze the substrate and produce 10 mg of reducing sugar per minute per milligram of protein at 37 °C. Lipase activity was also determined by a colorimetric method, and one unit of enzyme activity was defined as the amount of enzyme required to catalyze the release of 1 μmol of product per minute per milligram of protein at 37 °C. The protease activity was calculated and expressed according to the instructions provided with the corresponding assay kit.

### 2.8. Determination of Intestinal Gene Expression

According to the method described by Liu et al. [27], the expression levels of inflammation- and antioxidant-related genes in the intestinal tissues of coho salmon were determined by real-time quantitative PCR (RT-qPCR). The antioxidant-related genes analyzed included nuclear factor erythroid 2-related factor 2 (*nrf2*), Kelch-like ECH-associated protein 1 (*keap1*), glutathione reductase (*gsr*), glutathione S-transferase (*gst*), catalase (*cat*), glutathione peroxidase (*gpx*), and superoxide dismutase (*sod*). The inflammation-related genes analyzed included nuclear factor kappa B (*nf-κb*), tumor necrosis factor alpha (*tnf-α*), transforming growth factor beta 1 (*tgf-β1*), interferon gamma (*ifn-γ*), interleukin 1 beta (*il-1β*), interleukin 6 (*il-6*), and interleukin 8 (*il-8*). Specific primers were designed based on the coho salmon (*Oncorhynchus kisutch*) gene sequences available in the NCBI database. All primers were synthesized by Sangon Biotech Co., Ltd. (Shanghai, China), and *β-actin* was used as the reference gene. The primer sequences are listed in Table 3.

Total RNA was extracted using the SteadyPure Universal RNA Extraction Kit (Hunan Accurate Biotechnology Co., Ltd., Changsha, China), and RNA integrity, purity, and concentration were assessed by 2% agarose gel electrophoresis and a NanoDrop^®^ 2000 spectrophotometer. Subsequently, cDNA was synthesized from the extracted RNA using the Evo M-MLV Reverse Transcription Kit (Hunan Accurate Biotechnology Co., Ltd., Changsha, China) under the following conditions: 50 °C for 30 min, 95 °C for 5 min, and 5 °C for 5 min.

RT-qPCR was performed using the SYBR Green Pro Taq HS qPCR Kit (Hunan Accurate Biotechnology Co., Ltd., Changsha, China) on a LightCycler^®^ 96 real-time PCR system. The amplification protocol consisted of an initial denaturation at 95 °C for 30 s, followed by 40 cycles of 95 °C for 5 s and 60 °C for 30 s. A melting curve analysis was performed at the end of amplification. Relative expression levels of the target genes were calculated using the 2^−ΔΔCt^ method [28].

### 2.9. Bioinformatic Analysis of High-Throughput Sequencing Data

A total of 12 gut microbiota samples from juvenile coho salmon were subjected to high-throughput sequencing, including three samples from each treatment group: control (CK), low-dose (GL), medium-dose (GM), and high-dose (GH). Each sample represented a pooled intestinal content sample collected from one net cage. The sequencing data were analyzed using the online Omicsmart platform provided by Guangzhou Kidio Biotechnology Co., Ltd., Guangzhou, China.

Raw sequencing reads were first subjected to quality control to remove low-quality sequences and obtain high-quality, valid reads. Paired-end reads were then merged, filtered, and screened for chimeric sequences. Subsequently, clustering analysis was performed at 97% sequence similarity to generate operational taxonomic units (OTUs). The relative abundances of OTUs among different samples were statistically analyzed, and representative OTUs were taxonomically annotated using the RDP (Ribosomal Database Project) Classifier. α-diversity and β-diversity analyses were performed to evaluate differences in gut microbial community structure within and among samples, respectively. In addition, the composition and relative abundance of intestinal microbiota among treatment groups were compared at the phylum and order levels.

Furthermore, PICRUSt2 was used to predict the potential functions of the intestinal microbial communities, and the KEGG database was applied to infer the metabolic pathways potentially involved, thereby generating the corresponding functional abundance profiles. It should be noted that PICRUSt2 provides predictive functional inference based on taxonomic composition and does not directly measure microbial gene expression or metabolic activity.

### 2.10. Intestinal Histological Sectioning

Intestinal samples fixed in 4% paraformaldehyde were processed using a conventional paraffin-embedding procedure. Briefly, the samples were dehydrated through a graded ethanol series, cleared in xylene, embedded in paraffin, and sectioned into serial slices with a thickness of 4 μm. The sections were stained with hematoxylin and eosin (H&E) and then examined under a light microscope. For each intestinal segment, microscopic fields containing intact villi with a straight orientation were selected for image acquisition using a Motic microscopic imaging system (Motic Images Plus 2.0 software). Muscularis thickness (MT), villus length (VL), and villus width (VW) were then measured.

### 2.11. Statistical Analysis

Except for the high-throughput sequencing data of the gut microbiota, all other experimental data were analyzed using IBM SPSS Statistics 25 software. Prior to statistical analysis, all datasets were tested for normality and homogeneity of variance. When these assumptions were satisfied, differences among treatment groups were analyzed by one-way analysis of variance (one-way ANOVA), followed by Duncan’s multiple range test when a significant effect was detected (*p* < 0.05).

All results are presented as mean ± standard deviation (mean ± SD). All statistical analyses were conducted using the net cage as the experimental unit (*n* = 3). Analyses related to gut microbial diversity, community composition, and functional prediction were performed on the Omicsmart platform, and the corresponding statistical methods are described in the relevant figure and table legends.

## 3. Results

### 3.1. Effects of Dietary S. laevolacticus Supplementation on the Growth Performance

Compared with the control group (CK), dietary supplementation with *S. laevolacticus* significantly increased final body weight, weight gain rate (WGR), specific growth rate (SGR), and protein efficiency ratio (PER) in juvenile coho salmon (*p* < 0.05). Among the supplemented groups, the GM group showed the highest values for final body weight, WGR, SGR, and PER, all of which were significantly higher than those of the other groups (*p* < 0.05). In addition, the feed conversion ratio (FCR) was significantly reduced in the GL, GM, and GH groups compared with the CK group (*p* < 0.05). Notably, the GM group exhibited the lowest FCR, which was significantly lower than that of the GL and GH groups (*p* < 0.05) (Table 4).

No significant differences were observed among treatments in initial body weight, survival rate (SR), condition factor (CF), hepatosomatic index (HSI), or viscerosomatic index (VSI) (*p* > 0.05).

### 3.2. Effects of Dietary S. laevolacticus Supplementation on Digestive Enzyme Activities

Compared with the CK group, protease activity was significantly elevated in the GM and GH groups (*p* < 0.05). Among these, the GH group exhibited the highest protease activity, which was significantly higher than that of the CK and GL groups (*p* < 0.05). In addition, α-amylase activity in the GM and GH groups was significantly higher than that in the CK and GL groups (*p* < 0.05) (Table 5). In contrast, no significant differences were observed in lipase activity among the treatment groups (*p* > 0.05).

### 3.3. Effects of Dietary S. laevolacticus Supplementation on Serum Biochemical and Immune-Related Indices

As shown in Table 6, dietary supplementation with *S. laevolacticus* significantly increased serum albumin (ALB), lysozyme (LZM), and total protein (TP) levels, as well as acid phosphatase (ACP) and alkaline phosphatase (AKP) activities in the GL, GM, and GH groups compared with the CK group (*p* < 0.05). Among the supplemented groups, the GM group showed the highest ALB, LZM, and TP levels and the highest ACP and AKP activities, all of which were significantly higher than those in the GL and GH groups (*p* < 0.05).

For lipid-related parameters, serum HDL-C levels were significantly higher in all probiotic-supplemented groups than in the CK group (*p* < 0.05), with the highest value observed in the GL group. Serum LDL-C was significantly increased only in the GL group (*p* < 0.05), whereas no significant differences were detected between the GM or GH groups and the CK group (*p* > 0.05).

In addition, serum glucose (GLU), triglyceride (TG), and total cholesterol (T-CHO) levels were significantly lower in the GL, GM, and GH groups than in the CK group (*p* < 0.05). The GM group exhibited the lowest GLU and TG levels, while T-CHO levels were comparatively lower in the GM and GH groups and were both significantly lower than those in the CK group (*p* < 0.05).

### 3.4. Effects of Dietary S. laevolacticus Supplementation on Intestinal Morphology

**Figure 1:**
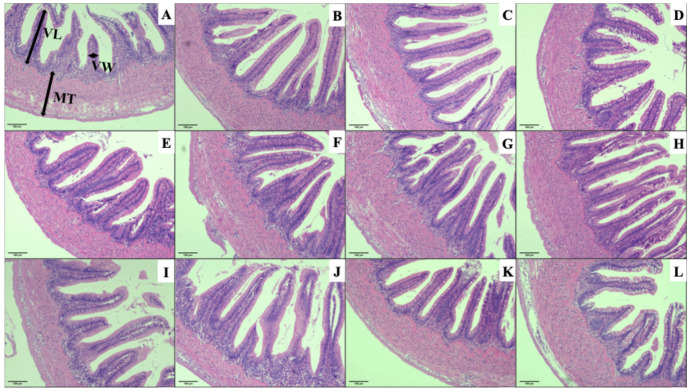
Effect of dietary *S. laevolacticus* supplementation on intestinal histology in juvenile coho salmon. (**A**): Foregut of CK group; (**B**): Foregut of GL group; (**C**): Foregut of GM group; (**D**): Foregut of GH group; (**E**): Midgut of CK group; (**F**): Midgut of GL group; (**G**): Midgut of GM group; (**H**): Midgut of GH group; (**I**): Hindgut of CK group; (**J**): Hindgut of GL group; (**K**): Hindgut of GM group; (**L**): Hindgut of GH group; MT: Muscularis thickness; VL: Villus length; VW: Villus width.

The results of intestinal histomorphology analysis are presented in Table 7 and Figure 1.

In the foregut, muscularis thickness, villus length, and villus width were all significantly increased in the GL, GM, and GH groups compared with the CK group (*p* < 0.05), with the GM group showing the most pronounced overall improvement.

In the midgut, muscularis thickness and villus length were significantly greater in the GL, GM, and GH groups than in the CK group (*p* < 0.05). Among these groups, the GM group exhibited the greatest muscularis thickness, whereas the GH group showed the longest villi. In contrast, villus width did not differ significantly among treatments (*p* > 0.05) (Table 7).

In the hindgut, muscularis thickness was significantly increased in the GM and GH groups relative to the CK group (*p* < 0.05), with the highest value observed in the GH group. Meanwhile, villus length was significantly higher in the GL, GM, and GH groups than in the CK group (*p* < 0.05), with the GL group showing the greatest value. No significant differences were detected in hindgut villus width among the treatment groups (*p* > 0.05) (Table 7).

### 3.5. Effects of Dietary S. laevolacticus Supplementation on the Expression of Intestinal Antioxidant- and Inflammation-Related Genes Expression

**Figure 2:**
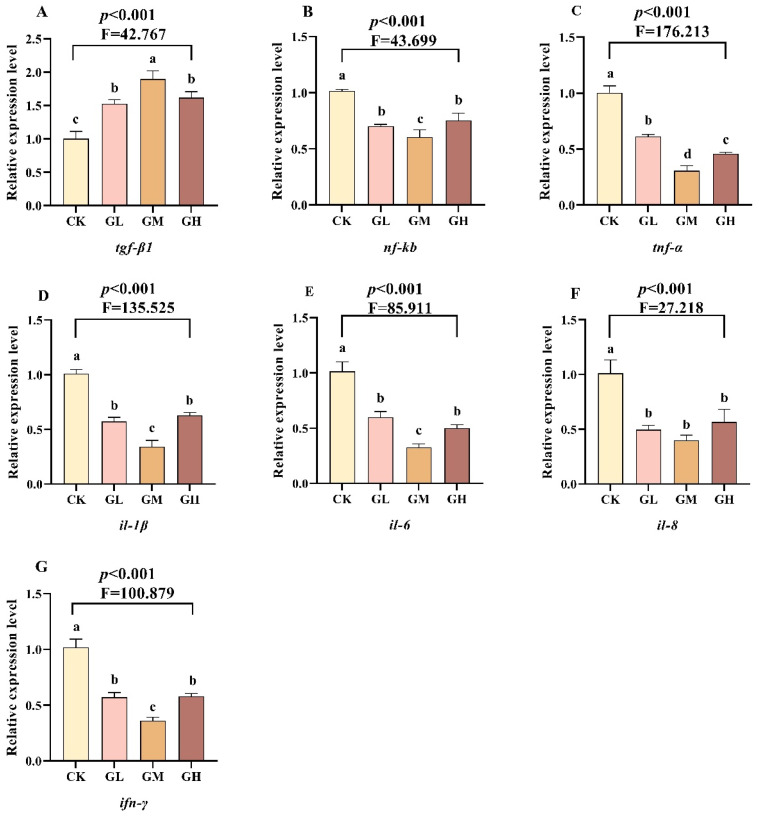
Effect of dietary *S. laevolacticus* supplementation on intestinal immune and inflammatory gene expression in juvenile coho salmon. All data above are expressed as mean ± standard deviation (*n* = 3), and different superscript letters indicate significant differences among groups (*p* < 0.05). (**A**): *tgf-β1*: Transforming growth factor beta-1. (**B**): *nf-κb*: Nuclear factor kappa B. (**C**): *tnf-α*: Tumor necrosis factor α. (**D**): *il-1β*: Interleukin-1β. (**E**): *il-6*: Interleukin-6. (**F**): *il-8*: Interleukin-8. (**G**): *ifn-γ*: Interferon γ.

As shown in Figure 2, the relative expression of *tgf-β1* in the intestine was significantly upregulated in the GL, GM, and GH groups compared with the CK group (*p* < 0.05), with the highest expression observed in the GM group.

At the same time, the relative expression levels of *nf-κb*, *tnf-α*, *ifn-γ*, *il-1β*, *il-6*, and *il-8* were significantly downregulated in all probiotic-supplemented groups compared with the CK group (*p* < 0.05). Among them, the GM group exhibited the lowest expression levels of *nf-κb*, *tnf-α*, *ifn-γ*, *il-1β*, and *il-6* (Figure 2).

**Figure 3:**
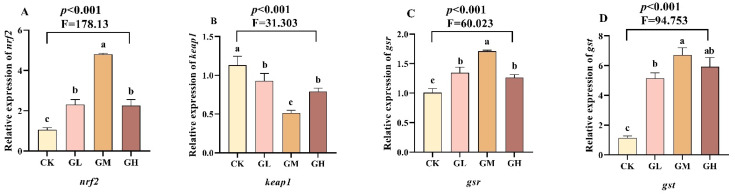

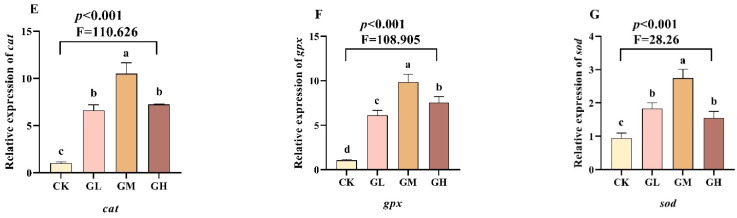
Effect of dietary *S. laevolacticus* supplementation on intestinal antioxidant gene expression in juvenile coho salmon. All data above are expressed as mean ± standard deviation (*n* = 3), and different superscript letters indicate significant differences among groups (*p* < 0.05). (**A**): *nrf2*: Nuclear factor erythroid 2-related factor 2. (**B**): *Keap1*: kelch-like ECH-associated protein 1. (**C**): *gsr*: Glutathione reductase. (**D**): *gst*: Glutathione S-transferase. (**E**): *cat*: Catalase. (**F**): *gpx*: Glutathione peroxidase. (**G**): *sod*: Superoxide dismutase.

As shown in Figure 3, the relative expression levels of the antioxidant-related genes *nrf2*, *gsr*, *gst*, *cat*, *gpx*, and *sod* were all significantly increased in the GL, GM, and GH groups compared with the CK group (*p* < 0.05), with the GM group showing the highest expression levels for most of these genes.

In contrast, intestinal keap1 expression was significantly reduced in the GL, GM, and GH groups relative to the CK group (*p* < 0.05), and the lowest keap1 expression was observed in the GM group (Figure 3).

### 3.6. Effects of Dietary S. laevolacticus Supplementation on Gut Microbial Diversity

**Figure 4:**
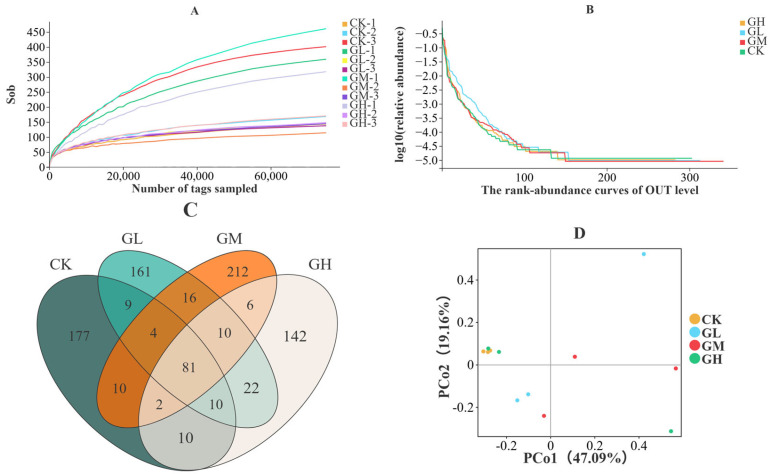
Effect of dietary *S. laevolacticus* supplementation on intestinal microbiota diversity in juvenile coho salmon. (**A**): Rarefaction curves. (**B**): Rank abundance curves. (**C**): Venn diagram of species. (**D**): PCoA scatter plot.

As shown in Figure 4A,B and Table 8, the rarefaction curves and rank-abundance curves of all treatment groups gradually approached a plateau, and Good’s coverage values were all greater than 0.999. These results indicate that the sequencing depth was sufficient and that the obtained data adequately reflected the composition of the intestinal microbial communities in juvenile coho salmon.

The numbers of OTUs identified in the CK, GL, GM, and GH groups were 303, 313, 341, and 283, respectively. A total of 81 OTUs were shared among all four groups, whereas the numbers of unique OTUs in the CK, GL, GM, and GH groups were 177, 161, 212, and 142, respectively (Figure 4C).

Analysis of α-diversity indices showed significant differences in the Shannon and Simpson indices among treatments (*p* < 0.05), although the patterns were not entirely consistent, with relatively higher values in the GL group and lower values in the GM group. In contrast, no significant differences were observed in the Chao1 index, ACE index, or Good’s coverage among the groups (*p* > 0.05) (Table 8).

Principal coordinates analysis (PCoA) of β-diversity revealed a certain degree of separation between the CK and GM groups in the two-dimensional ordination space, with PCo1 and PCo2 explaining 47.09% and 19.16% of the total variation, respectively (Figure 4D).

### 3.7. Effects of Dietary S. laevolacticus Supplementation on Gut Microbial Composition and Functional Potential

**Figure 5:**
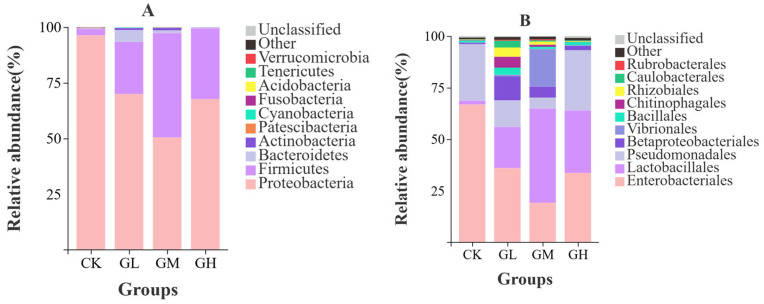
Effect of dietary *S. laevolacticus* on the intestinal microbiota structure of juvenile coho salmon. (**A**): Relative abundance at the phylum level; (**B**): Relative abundance at the order level.

At the phylum level, *Proteobacteria, Firmicutes*, and *Bacteroidota* were the dominant bacterial phyla in the intestine of juvenile coho salmon (Figure 5A). In the CK group, *Proteobacteria* were overwhelmingly predominant, accounting for 96.33% of the total relative abundance, whereas *Firmicutes* and *Bacteroidota* represented 2.80% and 0.29%, respectively. Following dietary supplementation with *S. laevolacticus*, the relative abundance of *Proteobacteria* declined overall, whereas that of *Firmicutes* and *Bacteroidota* increased accordingly. Among the probiotic-supplemented groups, the GM group showed the lowest relative abundance of *Proteobacteria* (50.43%) and the highest abundance of *Firmicutes* (46.91%) (Figure 5A).

At the order level, *Enterobacterales, Lactobacillales, Pseudomonadales*, and *Betaproteobacteriales* were the predominant bacterial taxa (Figure 5B). In the CK group, *Enterobacterales* accounted for the largest proportion (66.85%), while *Pseudomonadales* and *Betaproteobacteriales* represented 27.45% and 0.53%, respectively; together, these three orders comprised 94.83% of the total community. After *S. laevolacticus* supplementation, the relative abundances of *Enterobacterales*, *Pseudomonadales*, and *Betaproteobacteriales* decreased overall, with the GM group showing reductions to 19.15%, 5.31%, and 5.37%, respectively. In contrast, the relative abundance of *Lactobacillales* increased and reached its highest level in the GM group (45.67%) (Figure 5B).

**Figure 6:**
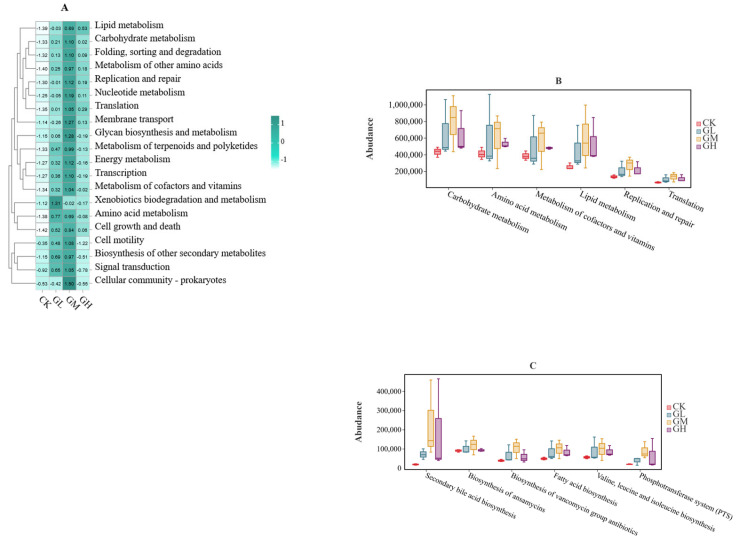
Effect of dietary *S. laevolacticus* supplementation on intestinal microbiota function in juvenile coho salmon. All data above are presented as mean ± standard deviation (*n* = 3), and distinct superscript letters denote significant differences among groups (*p* < 0.05). (**A**): Heat map of predicted functional abundance. (**B**): Differential analysis of predicted functions at KEGG level 2. (**C**): Differential analysis of predicted functions at KEGG level 3.

The results of functional prediction are shown in Figure 6. Compared with the CK group, the overall functional abundance of the intestinal microbiota showed an increasing trend in the probiotic-supplemented groups, with relatively higher values observed in the GM group (Figure 6A). At KEGG level 2, functional categories related to carbohydrate metabolism, amino acid metabolism, lipid metabolism, metabolism of cofactors and vitamins, replication and repair, and translation all showed upward trends; however, none of these differences reached statistical significance among treatments (*p* > 0.05) (Figure 6B). Similarly, at KEGG level 3, pathways including secondary bile acid biosynthesis, ansamycin biosynthesis, vancomycin-group antibiotic biosynthesis, fatty acid biosynthesis, branched-chain amino acid biosynthesis, and the phosphotransferase system (PTS) also exhibited increasing trends, although these changes were likewise not statistically significant (*p* > 0.05) (Figure 6C).

### 3.8. Correlation Analysis Among Growth Performance, Digestive Function, Intestinal Morphology, and Gut Microbiota

**Figure 7:**
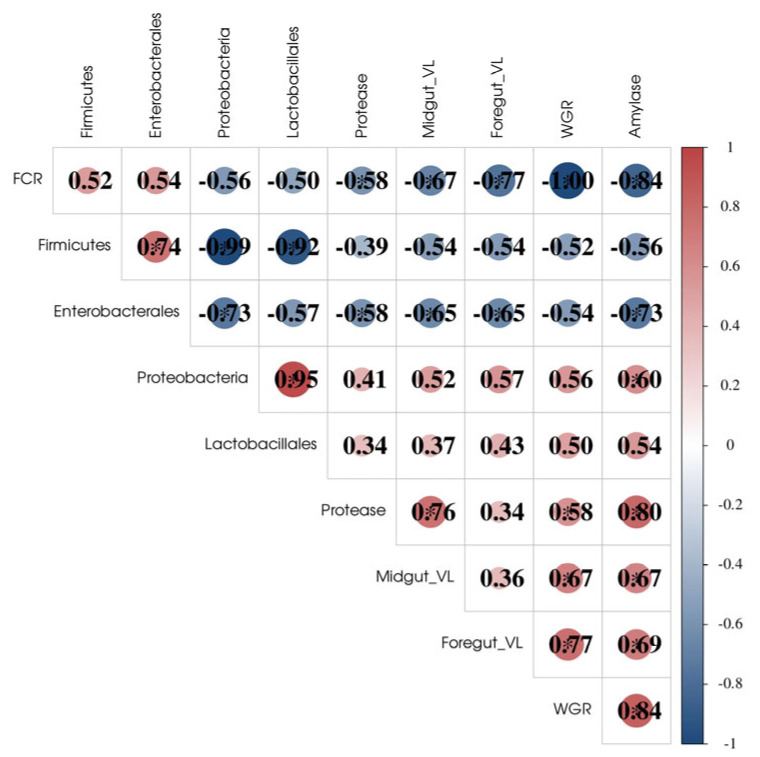
Correlation analysis among growth performance, digestive enzyme activities, intestinal morphology, and gut microbiota in juvenile coho salmon fed *S. laevolacticus*-supplemented diets. The lower triangle displays Spearman correlation coefficients, and the upper triangle represents correlation strength and direction by circle size and color (red, positive correlation; blue, negative correlation). Asterisks indicate statistically significant correlations (* *p* < 0.05; *n* = 12). WGR, weight gain rate; VL, villus length.

To further explore the potential relationships among growth performance, digestive function, intestinal morphology, and gut microbial composition, Spearman correlation analysis was conducted (Figure 7). The results showed that WGR was positively correlated with protease activity (r = 0.58), amylase activity (r = 0.84), midgut villus length (r = 0.67), and foregut villus length (r = 0.77). Protease activity was positively associated with midgut villus length (r = 0.76) and amylase activity (r = 0.80), suggesting coordinated enhancement of digestive function and intestinal development.

Regarding microbial parameters, *Enterobacteriales* showed negative associations with protease activity (r = −0.58), amylase activity (r = −0.73), midgut villus length (r = −0.65), foregut villus length (r = −0.65), and WGR (r = −0.54). In contrast, *Lactobacillales* exhibited positive correlations with WGR (r = 0.50), foregut villus length (r = 0.43), and amylase activity (r = 0.54), suggesting potential links between beneficial microbial taxa and host digestive and growth performance.

Several of the observed correlations reached statistical significance, particularly those involving digestive enzyme activities, villus development, and dominant microbial taxa. These findings suggest that the growth-promoting effects of *S. laevolacticus* may be linked to coordinated improvements in gut microbial composition, digestive enzyme activities, and intestinal villus development.

## 4. Discussion

### 4.1. S. laevolacticus Improves Growth Performance and Serum Biochemical Profiles

Growth performance parameters are widely recognized as key indicators for assessing the nutritional status of aquatic animals and the efficiency of feed utilization [29]. Among them, weight gain rate (WGR) and specific growth rate (SGR) directly reflect growth rate and growth outcome over the experimental period, whereas feed conversion ratio (FCR) and protein efficiency ratio (PER) are commonly used to evaluate feed conversion efficiency and protein utilization, respectively [30]. In general, increases in WGR, SGR, and PER together with a reduction in FCR indicate improved efficiency in nutrient utilization and are therefore regarded as important criteria for evaluating the effectiveness of feed additives [31]. In the present study, dietary supplementation with *S. laevolacticus* significantly increased final body weight, WGR, SGR, and PER, while significantly decreasing FCR, indicating that this strain markedly improved both growth performance and feed utilization in juvenile coho salmon. Among all treatments, the GM group exhibited the most favorable overall growth-promoting effect. Notably, no abnormal fluctuations were observed in survival rate, condition factor, hepatosomatic index, or viscerosomatic index among the treatment groups, suggesting that *S. laevolacticus* enhanced growth without causing obvious adverse effects on the general physiological condition of the fish. Previous reviews have shown that probiotic groups such as *Lactobacillus* and *Bacillus* are often associated with improved growth performance and feed utilization in aquatic animals [19]. The present findings are consistent with this general pattern and further support the application potential of *S. laevolacticus* as a dietary probiotic candidate.

In addition to growth performance, serum biochemical indices provide important information on nutritional metabolism and the overall physiological status of fish [32]. In this study, dietary supplementation with *S. laevolacticus* significantly increased serum total protein (TP) and albumin (ALB) levels, suggesting an improvement in protein nutritional status [33,34]. At the same time, serum lysozyme (LZM) content as well as acid phosphatase (ACP) and alkaline phosphatase (AKP) activities were significantly elevated, indicating an enhancement of nonspecific immune status following supplementation with this strain [35,36]. Similar findings have also been reported for lactic acid bacteria and spore-forming bacteria in other fish species [37,38], suggesting that probiotic supplementation may influence not only growth performance but also the overall physiological condition of the host. With respect to glucose and lipid metabolism, the present study further revealed a relatively consistent trend toward improvement. Serum HDL-C levels were significantly increased in all probiotic-supplemented groups, whereas LDL-C was elevated only in the low-dose group and did not differ significantly from the control in the medium- and high-dose groups. Meanwhile, GLU, TG, and T-CHO levels were all significantly reduced. Taken together, these changes suggest that dietary supplementation with *S. laevolacticus* contributed to a more favorable metabolic profile in juvenile coho salmon, particularly in terms of energy and lipid metabolism [39,40]. This result is comparable to previous findings obtained with Bacillus cereus in juvenile coho salmon [11], implying that spore-forming probiotic strains may share certain common benefits in improving metabolic status in this species. Overall, these results indicate that the effects of *S. laevolacticus* supplementation were not limited to improvements in growth-related traits but were also accompanied by coordinated enhancements in nutritional, metabolic, and immune-related physiological status.

### 4.2. S. laevolacticus Improves Digestive Enzyme Activities and Optimizes Intestinal Morphology

Digestive and absorptive capacity constitutes an essential physiological basis for growth and feed utilization efficiency in fish. Accordingly, changes in digestive enzyme activity and intestinal histological features are commonly used as important indicators for evaluating digestive and absorptive status [41]. In the present study, intestinal protease and α-amylase activities were significantly increased in the GM and GH groups, whereas lipase activity remained unchanged, suggesting that dietary supplementation with *S. laevolacticus* enhanced the capacity of juvenile coho salmon to digest proteins and carbohydrates [42]. Previous studies have shown that certain probiotic strains are capable of producing extracellular hydrolytic enzymes, including proteases and amylases, thereby improving the degradation efficiency of dietary substrates [43]. In addition, after colonizing the intestinal tract, probiotics may indirectly stimulate the secretion of endogenous digestive enzymes by improving the luminal microenvironment, maintaining epithelial function, and enhancing the activity of digestive tissues [15]. These findings suggest that the beneficial effects of probiotics on digestive function may be associated with both exogenous enzymatic contributions and enhanced digestive capacity of the host. Similar growth-promoting and digestion-enhancing effects have also been reported in other fish species; for example, supplementation with lactic acid bacteria or spore-forming bacteria improved growth performance and feed utilization in African catfish and Nile tilapia [44,45]. Taken together with the lower feed conversion ratio and higher protein efficiency ratio observed in this study, the enhancement of digestive enzyme activity was likely one of the major bases underlying the growth-promoting effect of *S. laevolacticus*.

In addition to digestive enzyme activity, improvements in intestinal morphology provide histological evidence for enhanced nutrient utilization [46]. In the present study, dietary supplementation with *S. laevolacticus* significantly increased villus length in the foregut, midgut, and hindgut, as well as muscularis thickness in specific intestinal segments, indicating that both absorptive structure and propulsive function of the intestine were improved to some extent. In general, greater villus development implies a larger absorptive surface area, whereas a thicker muscularis layer is conducive to more efficient transport and mixing of digesta. These structural changes may act synergistically with elevated digestive enzyme activities to facilitate nutrient digestion and absorption, thereby contributing to improved growth [15]. Similar observations have been reported in other fish probiotic studies. For example, *Lactobacillus plantarum* and *Lacticaseibacillus rhamnosus* were shown to enhance villus development and muscularis thickness in tilapia and other cultured fish species, thereby improving mucosal function and nutrient absorption efficiency [47,48]. From a mechanistic perspective, these morphological improvements may be related to the optimization of the intestinal nutritional environment and the alleviation of local irritation following probiotic supplementation. Previous studies suggest that probiotics can promote epithelial renewal and functional maintenance by participating in dietary substrate degradation, improving the intestinal microecological environment, and reducing inflammation-related stress, thereby creating more favorable local conditions for villus development and muscularis maturation [15,43].

It is worth noting that the degree of improvement in digestive enzyme activities and histological indices was not entirely consistent across the different dosage groups. For instance, although the high-dose group still showed improvements in certain enzyme activities and villus length parameters, its overall growth performance did not surpass that of the medium-dose group. This suggests that the growth-promoting effect of probiotics is not determined by any single index alone but is more likely the result of coordinated changes in digestive function, intestinal structure, local physiological status, and microbial community composition. The non-linear dose–response pattern, with the medium dose outperforming the high dose, may be explained by factors such as dose-dependent saturation, microbial competition in the gut, or host immune tolerance at excessive bacterial loads. Further studies are needed to elucidate the precise mechanisms underlying this observation.

### 4.3. S. laevolacticus Enhances Intestinal Antioxidant-Related Gene Expression and Suppresses Inflammation-Related Gene Expression

The intestine is not only a major site for nutrient absorption, but also a critical interface through which the host interacts with the external environment and the intestinal microbiota. Maintaining low oxidative stress and an appropriately regulated immune response is therefore essential for intestinal homeostasis [44,49]. From the perspective of antioxidant regulation, molecules associated with the Nrf2/Keap1 system are widely regarded as important indicators of the host antioxidant response [27]. In the present study, dietary supplementation with *S. laevolacticus* significantly upregulated the relative expression of *nrf2*, *gsr*, *gst*, *cat*, *gpx*, and *sod* in the intestine of juvenile coho salmon, while downregulating *keap1*. These results suggest that supplementation with this strain may enhance the transcriptional potential for antioxidant responses in the intestine. As the Nrf2/Keap1-associated regulatory system is considered a major basis for the cellular defense against oxidative stress, these findings further imply that the intestinal environment may have shifted toward a state more favorable for the maintenance of redox balance following supplementation [50,51]. Similar results have also been reported in previous studies, in which supplementation with lactic acid bacteria or spore-forming bacteria was accompanied by increased *nrf2* expression, decreased *keap1* expression, and enhanced expression of downstream antioxidant genes in fish [52,53].

From the perspective of inflammatory regulation, the present study showed that the expression levels of several pro-inflammatory genes, including *nf-κb*, *tnf-α*, *ifn-γ*, *il-1β*, *il-6*, and *il-8*, were all significantly reduced, whereas the expression of *tgf-β1* was significantly increased. The observed downregulation of *nf-κb* and several pro-inflammatory cytokine genes suggests that dietary *S. laevolacticus* supplementation may contribute to the attenuation of intestinal inflammatory responses. However, because only transcriptional responses were evaluated in the present study, direct involvement of the NF-κB signaling pathway requires further verification at the protein level. These results suggest that dietary supplementation with *S. laevolacticus* did not provoke excessive inflammatory activation; instead, it may have been associated with reduced transcriptional activity of pro-inflammatory genes, thereby shifting the local intestinal environment toward a lower inflammatory state and a more balanced pattern of immune regulation [54]. Previous studies have likewise shown that probiotic supplementation may be accompanied by reduced intestinal expression of pro-inflammatory cytokines and increased expression of anti-inflammatory or immunoregulatory factors in fish. For example, *Lactobacillus rhamnosus* GCC-3 has been reported to downregulate intestinal NF-κB signaling and alleviate intestinal inflammation in fish [55]. In another study, supplementation with lactic acid bacteria significantly reduced intestinal *il-1β*, *il-6*, and *tnf-α* expression while increasing *tgf-β1* expression, thereby contributing to the alleviation of local inflammation and the maintenance of intestinal homeostasis [56].

Taken together, the antioxidant- and inflammation-related results indicate that supplementation with *S. laevolacticus* did not merely alter a single category of response but rather shifted the intestinal environment of juvenile coho salmon toward a pattern characterized by “higher antioxidant capacity and lower inflammatory tone.” These coordinated changes may be associated with an improvement in the local intestinal environment following probiotic supplementation. On the one hand, the optimization of gut microbial structure and the reduction in potentially harmful microbial stimuli may help limit excessive ROS generation during inflammatory processes, thereby alleviating oxidative stress [57,58]. On the other hand, probiotic-derived metabolites and probiotic-mediated modulation of the local metabolic environment may also contribute to enhanced antioxidant gene expression and attenuated inflammatory responses [55,59]. When considered together with the improved intestinal morphology and elevated serum immune-related indices observed in this study, these findings suggest that dietary supplementation with *S. laevolacticus* promoted an intestinal state more conducive to homeostatic maintenance, thereby supporting growth improvement in juvenile coho salmon. Nevertheless, it should be noted that the present evaluation of antioxidant status was based solely on mRNA expression levels; biochemical indices such as SOD, CAT, GPx, GSH, T-AOC, and MDA were not measured in intestinal tissue. Therefore, the functional output of the observed transcriptional changes requires further validation at the protein and enzymatic activity levels.

### 4.4. S. laevolacticus-Mediated Remodeling of Gut Microbial Structure and Its Potential Functional Significance

The gut microbiota represents a key biological link connecting host nutrient metabolism, immune regulation, and overall health status. Changes in community composition often provide more meaningful insight into host functional responses than simple increases or decreases in diversity alone [60]. In fish, intestinal microorganisms influence growth performance and physiological homeostasis through their involvement in energy acquisition, nutrient transformation, mucosal development, immune responses, and disease resistance [61,62,63]. Therefore, evaluating probiotic effects from the perspectives of microbial composition and functional potential is important for understanding their physiological significance.

In the present study, dietary supplementation with *S. laevolacticus* caused only limited changes in α-diversity, and the trends among different diversity indices were not fully consistent, indicating that the probiotic effect was not necessarily reflected by a uniform increase or decrease in microbial diversity. In contrast, shifts in community composition were more informative. Principal coordinates analysis (PCoA) revealed a certain degree of separation between the CK and GM groups, suggesting that medium-dose supplementation exerted a relatively pronounced influence on intestinal microbial structure.

At the phylum level, supplementation with *S. laevolacticus* was associated with an overall reduction in the relative abundance of *Proteobacteria* and corresponding increases in *Firmicutes* and *Bacteroidota*, with the most evident shift observed in the medium-dose group. Previous studies have suggested that an abnormal increase in *Proteobacteria* is often associated with microbial imbalance or elevated potentially harmful stimulation [64], whereas *Firmicutes* contains many taxa related to fermentative metabolism and maintenance of intestinal homeostasis. Thus, the microbial changes observed in the present study generally point toward a community structure more favorable for maintaining intestinal health [65]. At the order level, the relative abundances of *Enterobacterales* and *Pseudomonadales* declined overall, whereas *Lactobacillales* increased, further indicating that dietary supplementation with *S. laevolacticus* shifted the intestinal microbiota in a direction potentially beneficial to host homeostasis. Notably, *Lactobacillales* was particularly enriched in the medium-dose group, and this change was mainly reflected at the genus level by increases in lactic acid bacteria-related taxa such as *Lactobacillus* and *Leuconostoc*. These compositional shifts, particularly the enrichment of *Lactobacillales*, are likely associated with increased production of beneficial metabolites such as lactic acid and short-chain fatty acids (SCFAs), which can lower intestinal pH, inhibit pathogenic bacteria, and modulate host immune and redox status.

Correlation analysis further supported the potential links between gut microbial modulation and host physiological responses. *Lactobacillales* showed positive associations with growth performance and digestive function, whereas *Enterobacteriales* were negatively correlated with digestive enzyme activities, villus development, and WGR. In addition, growth-related indices were positively associated with digestive enzyme activities and intestinal morphological parameters. Although these correlations do not establish direct causality, they provide further evidence that the beneficial effects of *S. laevolacticus* may involve coordinated regulation of gut microbial composition, digestive capacity, and intestinal development, ultimately contributing to improved growth performance.

At the functional level, PICRUSt2-predicted functional profiles showed trends toward enhanced carbohydrate and amino acid metabolism in the GM group, broadly paralleling the improved digestive capacity, reduced inflammatory tone, and enhanced antioxidant transcriptional potential observed in the host. This pattern is consistent with the overall superiority of the medium-dose group in terms of growth performance, serum biochemical indices, intestinal morphology, and gene expression responses.

Functional prediction using PICRUSt2 further indicated that supplementation with *S. laevolacticus* tended to increase microbial functions related to carbohydrate metabolism, amino acid metabolism, lipid metabolism, as well as replication and repair. Although these differences did not reach statistical significance, their directional trends were broadly consistent with the enhanced digestive capacity, improved metabolic status, and better growth performance observed in the host.

It should be noted that although strain GXF3206 exhibited no detectable hemolytic activity and showed favorable gastrointestinal tolerance and acute in vivo safety, the present study did not evaluate antibiotic resistance profiles, virulence-associated genes, or the potential risk of horizontal gene transfer. Therefore, further genomic and safety assessments are warranted before large-scale commercial application of this strain in aquaculture. In addition, strain identification in the present study was primarily based on 16S rRNA gene sequencing. Future studies incorporating phylogenetic analysis and whole-genome sequencing will be valuable for further confirming the taxonomic status and genomic characteristics of strain GXF3206. Because these functional profiles were inferred from PICRUSt2 prediction rather than directly measured, they should be regarded as potential functional trends rather than definitive evidence of microbial metabolic activity.

### 4.5. Integrated Mechanistic Interpretation

Taken together, the findings of the present study support an integrated mechanistic framework through which dietary *S. laevolacticus* supplementation promotes host performance and intestinal health in juvenile coho salmon. Specifically, supplementation with *S. laevolacticus* was associated with a shift in gut microbial composition characterized by a reduced relative abundance of Proteobacteria and an increased abundance of *Firmicutes* and lactic acid bacteria-related taxa. Such microbial remodeling may contribute to a more stable intestinal environment and improved microbial functional potential [66,67]. Concurrently, enhanced digestive enzyme activities and improved intestinal morphology suggest a greater capacity for nutrient digestion and absorption. These physiological improvements were accompanied by upregulation of antioxidant-related genes and downregulation of inflammation-related genes, indicating a more favorable intestinal status characterized by enhanced antioxidant defense and reduced inflammatory tone. This pattern aligns with previous reports on the beneficial effects of lactic acid bacteria and other probiotics in finfish aquaculture [68,69]. Collectively, these interconnected responses may act synergistically to improve nutrient utilization efficiency, maintain intestinal homeostasis, and ultimately promote growth performance in juvenile coho salmon. Nevertheless, the causal relationships among microbial modulation, intestinal physiology, and host growth remain to be fully elucidated and warrant further investigation through integrative multi-omics and functional validation approaches.

## 5. Conclusions

In summary, dietary supplementation with *S. laevolacticus* improved growth performance and feed utilization in juvenile coho salmon, particularly at 0.90 × 10^9^ CFU/g diet under the present experimental conditions. These benefits were accompanied by enhanced digestive enzyme activities, improved serum immune-related and biochemical indices, better intestinal morphology, altered antioxidant- and inflammation-related transcriptional responses, and shifts in gut microbial composition. However, this study has several limitations, including the evaluation of antioxidant and inflammatory status solely at the transcriptional level without protein-level validation, a single rearing environment, and the absence of a pathogen challenge trial to confirm disease resistance. Therefore, *S. laevolacticus* may serve as a promising probiotic candidate for coho salmon culture, although further studies are needed to validate its mechanisms, long-term safety, and practical application value.

## Figures and Tables

**Table 1:** Experimental dietary formulation (g/100 g of dry feed) and approximate composition (%, dry matter basis).

Ingredient	Content
Fish meal	40.00
Chicken powder	5.00
Shrimp powder	5.00
Soybean meal	15.00
Peanut meal	7.00
Flour	13.10
Starch	3.00
Fish oil	7.00
Soybean oil	2.50
Ca(H_2_PO_4_)_2_	1.00
Minerals premix ^1^	0.50
Vitamins premix ^2^	0.50
Choline chloride	0.30
Vitamin C	0.10
Approximate composition ^3^	
Crude protein (%)	44.70
Crude lipid (%)	14.30
Ash (%)	7.88
Moisture (%)	9.08
Gross energy (MJ/kg)	18.70

^1^ Composition of the mineral premix (mg/kg): AlK(SO_4_)_2_⋅12H_2_O, 123.7; CaCl_2_, 17,879.8; CuSO_4_⋅5H_2_O, 31.7; CoCl_2_⋅6H_2_O, 48.9; FeSO_4_⋅7H_2_O, 707.4; MgSO_4_⋅7H_2_O, 4316.8; MnSO_4_⋅4H_2_O, 31.1; ZnSO_4_⋅7H_2_O, 176.7; KCl, 1191.9; KI, 5.3; NaCl, 4934.5; Na_2_SeO_3_⋅H_2_O, 3.4; Ca(H_2_PO_4_)_2_⋅H2O, 12,457.0; KH_2_PO_4_, 9930.2. ^2^ Composition (IU or g/kg vitamin premix): retinal palmitate, 10,000 IU; cholecalciferol, 4000 IU; α-tocopherol, 75.0 IU; menadione, 22.0 g; thiamine HCl, 40.0 g; riboflavin, 30.0 g; D-calcium pantothenate, 150.0 g; pyridoxine HCl, 20.0 g; meso-inositol, 500.0 g; D-biotin, 1.0 g; folic acid, 15.0 g; ascorbic acid, 200.0 g; niacin, 300.0 g; cyanocobalamin, 0.3 g. ^3^ Approximate composition values were measured.

**Table 2:** Viable bacterial counts of *S. laevolacticus* in the experimental diets.

Index	Groups		
	GL	GM	GH
viable bacterial count (CFU/g diet)	0.89 × 10^7^	0.90 × 10^9^	0.87 × 10^11^

**Table 3:** Real-time quantitative PCR primers for genes of coho salmon.

Gene	Primer Sequence	GenBank	Tm (°C)	Size (bp)
*^1^ β-actin*	F: CCAAAGCCAACAGGGAGAA	XM_031822094.1	60	91
	R: AGGGACAACACTGCCTGGAT			
*^2^ nrf2*	F: TAGAGACGAGCAGCGAGCCAAG	XM_020461401.1	60	82
	R: GTTGAAGTCATCCACAGGCAGGTC			
*^3^ keap1*	F: CACACCGCCTCTCCTCCTCAG	XM_031799325.1	60	150
	R: GTTGGTTGGTGCCGTCGTAGC			
*^4^ gst*	F: CGCATTGACATGATGTGTGA	XM_031796997.1	60	121
	R: TGTCGAGGTGGTTAGGAAGG			
*^5^ gsr*	F: CCAGTGATGGCTTTTTTGAACTT	XM_020452987.2	60	61
	R: CCGGCCCCCACTATGAC			
*^6^ cat*	F: GCGTTCGGGTACTTTGAGGTGAC	XM_020456233.2	60	103
	R: TGGAGAAGCGGATGGCGATAGG			
*^7^ gpx*	F: GATTCGTTCCAAACTTCCTGCTA	XM_031789568.1	60	141
	R: GCTCCCAGAACAGCCTGTTG			
*^8^ sod*	F: CCGTTGGTGTTGTCTCCGAAGG	XM_020477154.2	60	101
	R: GAGGGTGACAATGCTCCAGTGAAG			
*^9^ nf-κb*	F: CAGCGTCCTACCAGGCTAAAGAGAT	XM_020502284.2	60	181
	R: GCTGTTCGATCCATCCGCACTAT			
*^10^ tnf-α*	F: GGCGAGCATACCACTCCTCT	XM_020497470.2	60	125
	R: TCGGACTCAGCATCACCGTA			
*^11^ tgf-β1*	F: CACCATGTCCACCTGTAAGTCTCTG	XM_020464995.2	60	111
	R: ATCTCTGGCTCCTTGGGCATCC			
*^12^ ifn-γ*	F: CAACATAGACAAACTGAAAGTCCA	XM_031819098.1	60	129
	R: ACATCCAGAACCACACTCATCA			
*^13^ il-1β*	F: GCGACATGGTGCGTTTCCTTTT	XM_020475860.2	60	129
	R: TGTCTACCGGTTTGGTGTAGTCCT			
*^14^ il-6*	F: GAGCTACGTAACTTCCTGGTTGAC	XM_020507339.2	60	134
	R: GCAAGTTTCTACTCCAGGCCTGAT			
*^15^ il-8*	F: ATCAGAATGTCAGCCAGCCTTGTC	XM_020486762.2	60	80
	R: CCCACGCCTCTCAGACTCATCC			

Note: ^1^ *β-actin*: Reference gene. ^2^ *nrf2*: Nuclear factor erythroid 2-related factor 2. ^3^ *keap1*: Kelch-like ECH-associated protein 1. ^4^ *gst*: Glutathione S-transferase. ^5^ *gsr:* Glutathione reductase. ^6^ *cat*: Catalase. ^7^ *gpx*: Glutathione peroxidase. ^8^ *sod*: Superoxide dismutase. ^9^ *nf-κb*: Nuclear factor kappa B. ^10^ *tnf-α*: Tumor necrosis factor. ^11^ *tgf-β1*: Transforming growth factor beta-1. ^12^ *ifn-γ*: Interferon gamma. ^13^ *il-1β*: Interleukin-1 beta. ^14^ *il-6*: Interleukin-6. ^15^ *il-8*: Interleukin-8.

**Table 4:** Effect of dietary *S. laevolacticus* on growth performance of juvenile coho salmon.

Index	Group				F-Value	*p*-Value
	CK	GL	GM	GH		
Initial weight (g)	130.53 ± 5.51	128.4 ± 8.05	127.77 ± 8.16	127.73 ± 9.65	0.082	0.968
Final weight (g)	273.33 ± 7.76 ^c^	299 ± 10.00 ^b^	325.67 ± 7.73 ^a^	299.33 ± 6.67 ^b^	25.841	<0.001
^1^ WGR (%)	109.50 ± 4.55 ^c^	133.20 ± 9.02 ^b^	155.35 ± 10.86 ^a^	135 ± 13.73 ^b^	11.356	0.003
^2^ SGR (%/d)	1.06 ± 0.03 ^c^	1.21 ± 0.01 ^b^	1.34 ± 0.06 ^a^	1.22 ± 0.08 ^b^	12.084	0.002
^3^ SR (%)	90.00 ± 5.00	90.00 ± 8.66	91.67 ± 14.43	91.67 ± 10.41	0.027	0.994
^4^ CF (%)	1.35 ± 0.17	1.29 ± 0.23	1.29 ± 0.02	1.27 ± 0.11	0.130	0.940
^5^ HSI (%)	1.17 ± 0.03	1.02 ± 0.13	1.16 ± 0.35	1.14 ± 0.32	0.250	0.859
^6^ VSI (%)	4.33 ± 0.79	4.28 ± 0.31	4.30 ± 0.38	4.22 ± 0.23	0.025	0.994
^7^ FCR	2.56 ± 0.09 ^a^	2.11 ± 0.13 ^b^	1.80 ± 0.13 ^c^	2.09 ± 0.20 ^b^	14.050	0.001
^8^ PER	0.89 ± 0.03 ^c^	1.08 ± 0.07 ^b^	1.26 ± 0.09 ^a^	1.10 ± 0.11 ^b^	11.356	0.003

Note: All data above are presented as mean ± standard deviation (*n* = 3). In the same row, data with different superscript letters indicate significant differences (*p* < 0.05). ^1^ WGR: Weight gain rate; ^2^ SGR: Specific growth rate; ^3^ SR: Survival rate; ^4^ CF: Condition factor; ^5^ HSI: Hepatosomatic index; ^6^ VSI: Viscerosomatic index; ^7^ FCR: Feed conversion ratio; ^8^ PER: Protein efficiency ratio.

**Table 5:** Effects of dietary *S. laevolacticus* on digestive enzyme activities of the juvenile coho salmon.

Index	Group				F-Value	*p*-Value
	CK	GL	GM	GH		
Protease (U/mg prot)	37.50 ± 3.80 ^c^	41.00 ± 4.00 ^bc^	48.80 ± 4.60 ^ab^	49.50 ± 4.20 ^a^	6.030	0.019
α-amylase (U/mg prot)	1.30 ± 0.10 ^b^	1.55 ± 0.15 ^b^	2.05 ± 0.16 ^a^	1.90 ± 0.13 ^a^	18.400	0.001
Lipase (U/mg prot)	9.00 ± 0.25	9.30 ± 0.45	8.95 ± 0.41	9.20 ± 0.50	0.476	0.708

Note: All data presented above are expressed as mean ± standard deviation (*n* = 3). In the same row, data with distinct superscript letters indicate significant differences (*p* < 0.05).

**Table 6:** Effect of dietary *S. laevolacticus* on serum physiological and biochemical indices of the juvenile coho salmon.

Index	Group				F-Value	*p*-Value
	CK	GL	GM	GH		
GLU ^1^ (mmol/L)	3.54 ± 0.13 ^a^	1.77 ± 0.02 ^c^	1.63 ± 0.09 ^c^	2.32 ± 0.22 ^b^	130.321	<0.001
HDL-C ^2^ (mmol/L)	5.70 ± 0.45 ^c^	10.55 ± 0.72 ^a^	8.60 ± 1.03 ^b^	8.29 ± 0.72 ^b^	20.744	<0.001
LDL-C ^3^ (mmol/L)	1.28 ± 0.06 ^b^	1.45 ± 0.04 ^a^	1.32 ± 0.02 ^b^	1.33 ± 0.01 ^b^	10.241	0.004
ALB ^4^ (g/L)	15.30 ± 0.14 ^d^	15.94 ± 0.32 ^c^	17.51 ± 0.28 ^a^	16.62 ± 0.17 ^b^	47.193	<0.001
TP ^5^ (mg/mL)	27.82 ± 0.16 ^c^	28.46 ± 0.72 ^c^	33.74 ± 0.05 ^a^	30.78 ± 0.18 ^b^	148.924	<0.001
TG ^6^ (mmol/L)	2.93 ± 0.05 ^a^	1.76 ± 0.12 ^b^	1.45 ± 0.08 ^c^	1.81 ± 0.1 ^b^	153.598	<0.001
T-CHO ^7^ (mmol/L)	6.50 ± 0.20 ^a^	5.41 ± 0.06 ^b^	4.55 ± 0.13 ^c^	4.39 ± 0.13 ^c^	148.704	<0.001
LZM ^8^ (μg/mL)	6.60 ± 0.23 ^d^	9.06 ± 0.27 ^c^	10.86 ± 0.43 ^a^	10.00 ± 0.32 ^b^	97.516	<0.001
ACP ^9^ (U/mL)	4.79 ± 0.23 ^c^	5.26 ± 0.17 ^bc^	6.54 ± 0.45 ^a^	5.40 ± 0.25 ^b^	19.338	0.001
AKP ^10^ (U/mL)	5.76 ± 0.17 ^b^	6.19 ± 0.13 ^b^	7.37 ± 0.32 ^a^	6.19 ± 0.22 ^b^	29.212	<0.001

Note: All data presented above are expressed as mean ± standard deviation (*n* = 3). In the same row, data with distinct superscript letters signify significant differences (*p* < 0.05). ^1^ GLU: Glucose; ^2^ HDL-C: High-density lipoprotein cholesterol; ^3^ LDL-C: Low-density lipoprotein cholesterol; ^4^ ALB: Albumin; ^5^ TP: Total protein; ^6^ TG: Triglyceride; ^7^ T-CHO: Total cholesterol; ^8^ LZM: Lysozyme; ^9^ ACP: Acid phosphatase; ^10^ AKP: Alkaline phosphatase.

**Table 7:** Effect of dietary *S. laevolacticus* supplementation on intestinal histology in juvenile coho salmon.

Index	Group				F-Value	*p*-Value
	CK	GL	GM	GH		
MT ^1^ (μm)						
Foregut	183.52 ± 2.55 ^d^	206.06 ± 2.94 ^c^	255.37 ± 5.72 ^a^	223.27 ± 6.91 ^b^	58.795	<0.001
Midgut	167.47 ± 2.62 ^d^	213.09 ± 9.11 ^c^	278.38 ± 3.29 ^a^	240.85 ± 12.82 ^b^	658.974	<0.001
Hindgut	172.86 ± 4.74 ^c^	180.74 ± 2.38 ^c^	199.50 ± 10.10 ^b^	235.36 ± 6.14 ^a^	455.204	<0.001
VL ^2^ (μm)						
Foregut	269.33 ± 2.54 ^c^	469.69 ± 12.63 ^a^	487.47 ± 4.80 ^a^	369.02 ± 7.20 ^b^	150.719	<0.001
Midgut	343.69 ± 12.61 ^b^	426.65 ± 20.14 ^a^	446.37 ± 7.59 ^a^	449.42 ± 12.52 ^a^	17.849	0.001
Hindgut	353.59 ± 6.40 ^c^	453.04 ± 10.39 ^a^	427.07 ± 4.74 ^b^	402.50 ± 4.46 ^b^	45.811	<0.001
VW ^3^ (μm)						
Foregut	65.74 ± 1.73 ^c^	83.53 ± 2.23 ^b^	94.77 ± 2.89 ^a^	85.70 ± 2.14 ^ab^	37.536	<0.001
Midgut	82.83 ± 7.27 ^a^	90.15 ± 4.04 ^ab^	88.56 ± 4.87 ^b^	87.90 ± 2.64 ^b^	4.593	0.038
Hindgut	74.85 ± 5.42 ^a^	87.25 ± 3.12 ^a^	82.15 ± 4.91 ^a^	86.63 ± 3.99 ^a^	2.773	0.111

Note: All data presented above are expressed as mean ± standard deviation (*n* = 3). In the same row, data with distinct superscript letters signify significant differences (*p* < 0.05). ^1^ MT: Muscularis thickness; ^2^ VL: Villus length; ^3^ VW: Villus width.

**Table 8:** Alpha diversity outcomes of the intestinal microbiota.

Index	Group				F-Value	*p*-Value
	CK	GL	GM	GH		
Shannon index	2.62 ± 0.17 ^ab^	3.04 ± 0.44 ^a^	2.18 ± 0.21 ^b^	2.54 ± 0.38 ^ab^	6.730	0.034
Simpson index	0.69 ± 0.05 ^ab^	0.77 ± 0.06 ^a^	0.60 ± 0.03 ^b^	0.70 ± 0.09 ^ab^	7.010	0.031
Chao index	288.91 ± 67.35	250.68 ± 78.63	285.44 ± 113.64	257.54 ± 56.61	0.031	0.458
Ace index	292.62 ± 72.59	255.92 ± 81.83	293.43 ± 117.9	263.97 ± 61.66	1.610	0.469
Coverage	0.9994 ± 0.0009	0.9996 ± 0.00008	0.9996 ± 0.00014	0.9995 ± 0.00004	0.420	0.740

Note: All data presented above are expressed as mean ± standard deviation (*n* = 3). In the same row, data with distinct superscript letters signify significant differences (*p* < 0.05).

## Data Availability

The data presented in this study are available on request from the corresponding author.
